# Chamber–Specific Molecular Profiling of Doxorubicin-Induced Cardiotoxicity via Integrated Multi-Omics Analysis

**DOI:** 10.3390/metabo16100746

**Published:** 2026-10-04

**Authors:** Jian Zhang, Yue Sun, Xiaozhen He, Yerui Zhang, Shijun Wang, Leilei Cheng

**Affiliations:** 1 Department of Cardiology, Zhongshan Hospital, Fudan University, Shanghai 200032, China; 2 Institute of Cardio-Oncology, Fudan University (in preparation), Shanghai 200032, China; 3 State Key Laboratory of Cardiovascular Diseases, Zhongshan Hospital, Fudan University, Shanghai 200032, China; 4 Shanghai Institute of Cardiovascular Diseases, Shanghai 200032, China; 5 Department of Echocardiography, Zhongshan Hospital, Fudan University, Shanghai 200032, China; 6 Zhongshan Hospital and Institute of Biomedical Sciences, Fudan University, 180 Fenglin Road, Shanghai 200032, China

**Keywords:** doxorubicin deposition, cardiac chamber specificity, phosphorylation remodeling, cardiac energy metabolism, multi–omics profiling

## Abstract

**Background**: Doxorubicin (Dox) induces dose-dependent Dox–induced cardiotoxicity (DIC), but the chamber-specific molecular mechanisms remain unclear, as traditional analyses rely on whole-heart homogenates. **Methods**: We established rat acute single–dose and chronic cumulative–dose DIC models, and separated left atrium (L–atrium) and left ventricle (L–ventricle) tissues for subsequent detection. Liquid chromatography–tandem mass spectrometry (LC–MS/MS) was used to quantify regional Dox tissue and serum concentrations. Histological HE and Masson’s staining were used to evaluate myocardial structural injury and collagen deposition. Integrated chamber-specific bulk proteomics, phosphoproteomics, and untargeted metabolomics were applied to systematically compare molecular perturbations between the L-atrium and L-ventricle. Two–way factorial statistical decomposition coupled with functional enrichment analysis was subsequently performed to identify both shared and chamber-specific molecular signatures. **Results**: Histological staining confirmed pronounced obvious myocardial fibrosis in both the L–atrium and L–ventricle under chronic Dox exposure. LC–MS/MS revealed prominent L–atrium–predominant Dox accumulation in the acute model, while inter–chamber drug distribution differences vanished after long–term repeated administration. Proteomic and metabolomic responses exhibited high inter–chamber consistency (r = 0.864 and r = 0.911, respectively). In contrast, the phosphoproteome was the dominant regulatory layer driving chamber divergence. A total of 14 core phosphosites with significant tissue–drug interaction effects were identified as key molecular switches. **Conclusions**: Our findings reveal preferential atrial Dox accumulation and distinct atrial phosphorylation remodeling following Dox exposure. The identified differential phosphosites serve as promising candidate targets for chamber-specific cardioprotection against DIC, pending further functional validation.

## 1. Introduction

Doxorubicin (Dox) is a highly effective anthracycline chemotherapeutic agent that has significantly improved survival rates for a wide spectrum of cancers. However, its clinical utility is severely limited by a dose-dependent cardiotoxicity, which can manifest as acute injury or chronic, progressive cardiomyopathy that ultimately leads to heart failure [1,2,3,4,5]. This life-threatening complication profoundly impacts the long-term quality of life and survival of cancer survivors, underscoring the urgent need to elucidate the underlying molecular mechanisms to develop effective cardioprotective strategies.

Dox exhibits high affinity for cardiac tissue, and previous studies have demonstrated that Dox and its metabolites accumulate within the myocardium. Following administration, Dox rapidly penetrates cardiomyocytes and interacts with cardiolipin, a lipid predominantly located on the inner mitochondrial membrane. This molecular interaction facilitates the passive diffusion of Dox into cardiomyocytes [6]. The pathogenesis of Dox–induced cardiotoxicity (DIC) is complex and multifactorial. Decades of research have implicated several key cellular processes, including oxidative stress generation, topoisomerase II–β inhibition, mitochondrial dysfunction, and induction of apoptosis and ferroptosis [7,8,9,10]. Temporally, DIC is divided into acute and chronic subtypes. Acute cardiotoxicity occurs within one week after administration, with an incidence of approximately 11%. Chronic cardiotoxicity may emerge months to years after treatment discontinuation [11,12,13]. Despite these advances, a critical gap remains in our spatial understanding of DIC. The vast majority of preclinical studies have relied on the analysis of homogenized whole–heart tissues. This approach fundamentally overlooks the profound anatomical, embryological, and functional heterogeneity among distinct cardiac compartments, such as the atria, ventricles, valves, and major vessels. Consequently, it is unknown whether Dox accumulation and the ensuing molecular perturbations occur uniformly across the heart or exhibit region–specific patterns. This hinders identification of the most vulnerable cardiac regions and the precise initial molecular events that trigger the cascade toward a terminal cardiac dysfunction, ultimately limiting the specificity of potential therapeutic interventions.

We hypothesize that the cardiac response to Dox is characterized by significant spatial and temporal heterogeneity, thereby conferring chamber–specific molecular vulnerabilities and response mechanisms that ultimately drive the progression of toxicity. To test this hypothesis, a strategy capable of capturing both the dynamic evolution and the spatial complexity of DIC is required. Here, we employed an integrative chamber–specific bulk multi–omics approach, which provides an unbiased and systems–level molecular profiling of specific heart regions. This study is designed to systematically investigate DIC by integrating precise quantification of Dox distribution via liquid chromatography–tandem mass spectrometry (LC-MS/MS), and complementary profiling of the proteome, metabolome, and phosphoproteome.

Recent cardiac multi–omics studies have shown that protein and metabolite layers can define disease–related functional modules in human heart failure [14]. The same logic can be applied to drug response. Here we used proteomics, phosphoproteomics and untargeted metabolomics to compare Dox–treated and control rat atrial and ventricular samples. The study was designed to answer three questions. First, which molecular changes are shared between the atrium and ventricle after Dox treatment? Second, which omics layer best separates the two chamber responses? Third, which candidate proteins, phosphosites and metabolites should be prioritized for validation?

## 2. Materials and Methods

### 2.1. Rat Model of Acute and Chronic DIC

An animal model of DIC was established in male Sprague–Dawley (SD) rats (body weight 200–220 g, 6–8 weeks old). Animals were randomly assigned to the acute injury group (*n* = 6), chronic accumulation group (*n* = 8), and corresponding age-matched control groups using simple randomization. All rats were housed under standard laboratory conditions: temperature 22 ± 2 °C, relative humidity 50 ± 5%, with a 12 h light–dark cycle. Animals had ad libitum access to standard rodent chow and drinking water. Rats in the acute injury group received a single intraperitoneal injection of 15 mg/kg doxorubicin hydrochloride (MCE, Shanghai, HY–15142), while those in the chronic accumulation group received weekly injections of 2.5 mg/kg for 6 consecutive weeks (cumulative dose: 15 mg/kg) [15,16,17]. Age–matched control rats received equivalent volumes of sterile saline via the same injection schedule. Animals from the acute and chronic groups were euthanized at 24 h after a single injection and at 1 week after the final dose, respectively. Serum was collected post–euthanasia, and the heart was dissected into the left atrium (L–atrium), left ventricle (L–ventricle), aortic root, and mitral–aortic valves.

### 2.2. Histological Analysis

After cardiac tissue dissection, tissue portions prepared for 5 μm coronal sections were fixed in 4% paraformaldehyde for 48 h and paraffin embedded, while the remaining tissue aliquots were frozen in liquid nitrogen and subjected to multi–omics analysis. For histological evaluation, sections were stained with hematoxylin and eosin (HE) to assess general myocardial architecture, including cardiomyocyte morphology, nuclear integrity, and inflammatory infiltration. To evaluate collagen deposition and fibrosis, Masson’s staining was performed, wherein collagen fibers were stained blue, cardiomyocytes red, and nuclei dark blue. All stained sections were examined under a light microscope by two independent investigators blinded to the experimental groups.

### 2.3. Quantification of Doxorubicin by LC-MS/MS

Approximately 10 mg tissue was homogenized in 100 μL normal saline, while 100 μL serum was directly used. A total of 15 μL of 1000 ng/mL daunorubicin internal–standard solution was spiked into each sample; mixtures were vortexed and stood for 10 min. Then, 250 μL acetonitrile was added for protein precipitation, followed by 1 min vigorous vortexing and centrifugation (14,000× *g*, 4 °C, 10 min). Supernatants were loaded into vials for LC–MS/MS analysis. Calibration series (1, 10, 50, 100, 500 ng/mL doxorubicin) were prepared by serial dilution in 50% acetonitrile with constant internal–standard concentration.

Separation was performed on a Vanquish Flex UHPLC with Waters (Milford, MA, USA) BEH Amide column (2.1 mm × 100 mm). Mobile phases were 0.1% formic acid–water and 40% acetonitrile /60% methanol. Gradient: 10–40% B (0–3 min); 40–95% B (3–5 min); 95–100% B (5–10 min); hold 100% B (10–10.5 min); return to 10% B and equilibrate to 14 min; flow rate 0.5 mL/min.

Detection was performed on TSQ Altis Plus triple-quadrupole MS under ESI^+^-MRM mode. Settings: spray voltage 3500 V; ion transfer tube 325 °C; sheath gas 50 arb; aux gas 10 arb. MRM transitions: doxorubicin 544.1 → 321.0 (29 V), 544.1 → 361.0 (27 V); daunorubicin 528.1 → 305.9 (51 V), 528.1 → 321.0 (27 V). Data acquisition and quantification were achieved via Xcalibur software (version 4.3, Thermo Fisher Scientific, USA). Doxorubicin concentrations were determined by internal-standard calibration against the established standard curve.

### 2.4. Experimental Procedures for Proteomics and Phosphoproteomics

#### 2.4.1. Protein Extraction

Serum samples retrieved from −80 °C storage were thawed on ice, followed by centrifugation at 15,000× *g* for 10 min under 4 °C. After discarding the pellet, the resulting supernatant was collected into fresh centrifuge tubes for depletion of high-abundance proteins, which was performed following the manufacturer’s protocol of the Pierce™ Top 14 Abundant Protein Depletion Spin Columns Kit (Thermo Scientific, Waltham, MA, USA). Protein concentrations were subsequently quantified via the BCA assay.

#### 2.4.2. Enzyme Digestion

##### Tandem Mass Tag (TMT) for Proteomics

Equivalent amounts of peptides from each sample were vacuum freeze–dried and reconstituted in 100 mM HEPES. Isobaric labeling was carried out according to the manufacturer’s instructions. In brief, labeling reagents were equilibrated to room temperature, dissolved in 100% acetonitrile, and mixed with peptide samples for 1 h at room temperature. Following labeling, equal amounts of peptides from all channels underwent spectrometry for labeling efficiency assessment. Upon passing quality control, 5% hydroxylamine was added to quench the reaction for 15 min. The labeled peptide pool was then desalted, vacuum-lyophilized, and stored for later use.

##### HPLC Fractionation for Proteomics

Peptides were fractionated by high–pH reversed–phase HPLC on an Agilent (Santa Clara, CA, USA)300 Extend C18 column (3.5 μm, 4.6 × 250 mm). Mobile phases were 2% acetonitrile (pH 9.0, A) and 98% acetonitrile (pH 9.0, B). Separations were carried out at 40 °C with UV detection at 214 nm. The gradient was as follows: 6% B (0–5 min); 6–8% B (5–8 min); 8–32% B (8–54 min); 32–95% B (54–60 min); 95% B (60–65 min); 95–5% B (65–70 min); 5–95% B (70–75 min); 95% B (75–80 min); 95–5% B (80–83 min); 5% B (83–90 min). The flow rate was 1 mL/min. Fractions were collected from 9 to 57 min (48 fractions in total), then combined into 16 pools and vacuum-dried until use.

#### 2.4.3. Affinity Enrichment for Phosphoproteomics

Resuspend the peptide sample in loading buffer (2% TFA, 70% ACN) and ensure complete dissolution. Mix with TiO_2_ beads and incubate on a rotating shaker for 30 min. Centrifuge at 800× *g* for 1 min at room temperature and collect the TiO_2_ beads. Wash the beads sequentially with Buffer A (0.5% TFA, 40% ACN) and Buffer B (0.4% TFA, 80% ACN). Elute the phosphorylated peptides using an elution buffer (15% ammonia, 40% ACN), then dry the eluate under vacuum and desalt the sample for further use.

#### 2.4.4. LC-MS/MS Analysis

##### DDA

Chromatographic separation was performed on an Easy–nLC 1200 UHPLC coupled to a Q Exactive HF–X mass spectrometer. Mobile phases A (0.1% formic acid + 2% acetonitrile in water) and B (0.1% formic acid + 80% acetonitrile in water) were used for peptide separation. Peptides (1 μg) were loaded onto a 20 cm column packed with 1.9 μm ReproSil–Pur C18 resin (Dr. Maisch GmbH, Ammerbuch, Germany). The gradient program: 0–26 min, 7–22% B; 26–34 min, 22–32% B; 34–37 min, 32–80% B; 37–40 min, 80% B; flow rate 450 nL/min.

Peptides were ionized using the NanoFlex ion source with spray voltage 2.1 kV. Full–MS scans covered *m*/*z* 400–1200 (resolution 60,000), and MS2 spectra were obtained at resolution 15,000. DDA mode selected the top 20 precursors for HCD fragmentation. AGC target was 5 × 10^4^, signal threshold 1 × 10^4^, maximum injection time 22 ms. Dynamic exclusion was set to 30 s to avoid repeated sampling of high-abundance peptide ions.

##### Astral DIA

For DIA measurement, peptides were chromatographically separated on a nanoflow Vanquish Neo UHPLC (Thermo Fisher Scientific) and detected on an Astral high–resolution mass spectrometer (Thermo Scientific) under positive–ion mode. Precursor full scans covered *m*/*z* 380–980 with 240,000 resolution (at *m*/*z* 200), normalized AGC target of 500%, and maximum IT of 5 ms. DIA–MS2 was acquired using 150 isolation windows of 4 *m*/*z* width; HCD fragmentation energy was set to 25 eV, with normalized AGC target of 500% and maximum IT of 10 ms.

DDA measurements were performed on an Easy–nLC 1200 UHPLC coupled to a Q Exactive HF–X mass spectrometer employing mobile phases A (0.1% formic acid, 2% acetonitrile in water) and B (0.1% formic acid, 80% acetonitrile in water). One microgram of reconstituted peptides was loaded onto a self–packed 20 cm column (1.9 μm ReproSil-Pur C18, Dr. Maisch GmbH). Elution gradient: 0–26 min 7–22% B; 26–34 min 22–32% B; 34–37 min 32–80% B; 37–40 min 80% B; flow rate 450 nL/min. Peptide ionization was achieved by the NanoFlex ion source (spray voltage 2.1 kV). Full–MS scan: *m*/*z* 400–1200, resolution 60,000; MS2 resolution 15,000. The top 20 precursors were subjected to HCD fragmentation. AGC target 5 × 10^4^, signal threshold 1 × 10^4^, maximum injection time 22 ms, and dynamic exclusion 30 s.

### 2.5. Experimental Procedures for Untargeted Metabolomics

#### 2.5.1. Sample Preparation for Serum

1. Take serum from the −80 °C freezer and place it on ice or in a 4 °C refrigerator. After the serum is completely thawed, vortex for 10 s, centrifuge at 12,000× *g*, 4 °C for 1 min, then place it on ice for later use.
2. PQC sample preparation: Mix 10 μL from each serum sample to create a Pool QC (PQC) sample, vortex to mix well.
3. SST (System Suitability Test) sample preparation: dilute the internal standard working solution five times with a 10% methanol/90% water solution (standard solution) as the SST sample.
4. Blank solution: Use extraction solvent as a blank solution (methanol pre-cooled in a −80 °C freezer for more than half an hour).
5. Take 20 μL serum, 20 μL PQC, and 20 μL blank solution each in a 2 mL centrifuge tube, add 20 μL internal standard working solution, then add 110 μL extraction solvent (methanol pre-cooled in a −80 °C freezer for more than half an hour), vortex and shake for 1 min; place at −20 °C for at least half an hour.
6. Centrifuge at 14,000× *g*, 4 °C for 10 min, transfer the supernatant to a new 2 mL centrifuge tube, vacuum freeze-dry, resuspend in 100 μL of 10% methanol/90% water solution (standard solution), vortex for 30 s, sonicate for 1 min, centrifuge at 14,000× *g*, 4 °C for 10 min, transfer the supernatant to a sample vial for mass spectrometry analysis.

#### 2.5.2. Sample Preparation for Tissue

Frozen serum samples (−80 °C) were thawed on ice or at 4 °C. Upon complete thawing, samples were vortexed for 10 s and centrifuged (12,000× *g*, 4 °C, 1 min), then maintained on ice. Pooled QC (PQC) was prepared by combining 10 μL from each serum sample and mixed by vortexing. A System Suitability Test (SST) sample was generated by 5–fold dilution of internal–standard working solution with 10% methanol/90% water. Pre–cooled methanol (stored at −80 °C for ≥30 min) served as the blank extraction solvent.

A total of 20 μL of serum, PQC and blank solvent were pipetted into individual 2 mL centrifuge tubes. After adding 20 μL internal–standard working solution and 110 μL cold extraction solvent, tubes were vortex–shaken for 1 min and incubated at −20 °C for ≥30 min. Samples were centrifuged at 14,000× *g* for 10 min at 4 °C; supernatants were harvested, vacuum–lyophilized, and redissolved in 100 μL 10% methanol–90% water. Following 30 s vortex and 1 min sonication, another round of centrifugation (14,000× *g*, 4 °C, 10 min) was carried out, and supernatants were transferred to sample vials for LC–MS analysis.

#### 2.5.3. LC–MS Conditions

Two complementary chromatographic setups coupled with positive– and negative–ion electrospray ionization were utilized to maximize metabolite coverage for untargeted metabolomics.

RP–C18 separation was performed on a Vanquish Flex UPLC using a Waters ACQUITY BEH C18 column (1.7 μm, 2.1 mm × 100 mm) at 40 °C with 5 μL injection volume. Mobile phases: A, 0.1% formic acid in water; B, 0.1% formic acid in acetonitrile/methanol (40/60, *v*/*v*). Gradient (0.5 mL/min): 0–0.5 min 95% B; 0.5–7 min 95–65% B; 7–8 min 65–40% B; 8–9 min 40% B; 9–9.1 min 40–95% B; 9.1–12 min 95% B.

High–polarity metabolites were separated on a Waters BEH Amide HILIC column (1.7 μm, 2.1 mm × 100 mm). Column temperature and injection volume were consistent with RP–C18 conditions. Mobile phase A contained 25 mM ammonium acetate and 25 mM ammonia water in water; mobile phase B was pure acetonitrile. The elution gradient was the same as for RP–C18.

All MS data were collected on a Q Exactive HF-X mass spectrometer equipped with an HESI source in positive– and negative–ion ddMS2 mode. Full–scan settings: resolution 60,000, AGC target 4 × 10^6^, maximum injection time 100 ms, *m*/*z* 60–900. ddMS2: top–20 precursors, resolution 15,000, AGC target 2 × 10^6^, maximum injection time 25 ms, stepped collision energies of 10, 40, 80 eV. Metabolite annotation was implemented via R packages XCMS and Spectra by searching against MoNA, GNPS, HMDB, MS-Dial LipidBlast and an in-house metabolite database.

### 2.6. Bioinformatics Analysis for Untargeted Metabolomics

#### 2.6.1. Quality Control of Data

Pooled QC (PQC) samples were produced by pooling sample extracts to evaluate reproducibility under uniform sample–processing conditions. Known–concentration internal standards were added to each sample to monitor detection stability. Less variation in internal–standard signal responses reflects more stable instrument performance and improved data quality.

#### 2.6.2. Sample Reproducibility Test

The coefficient of variation (CV), defined as standard deviation divided by the mean, was calculated on log_2_–transformed raw data to assess data dispersion. Cumulative–distribution curves illustrated CV–value frequencies; smaller overall CVs denoted improved experimental stability and quantitative reproducibility.

PCA was performed on log_2_–transformed data with RStudio (version 2023.06.0+421, Posit Software, PBC, Boston, MA, USA)’s native prcomp function using scale = TRUE for normalization. Results were visualized using scores derived from the top two principal components.

Sample–wise linear associations were quantified by Pearson correlation coefficients computed on log_2_–transformed intensities. The base–R cor() function with use = “pairwise.complete.obs” handled missing values, and correlation matrices were plotted as heatmaps with the Pheatmap R package (version 1.0.12).

#### 2.6.3. Metabolite Annotation and Classification

All measured metabolites were annotated, functionally characterized and categorized by querying HMDB and KEGG pathway databases. Visual outputs were produced using ggplot2 R package (v3.4.4).

#### 2.6.4. Differential Metabolite Analysis

Univariate and multivariate statistics were jointly applied to detect differential metabolites from log_2_–transformed raw data. Two–group mean differences were evaluated using R’s *t*-test function (*p* < 0.05 for significance). OPLS–DA was conducted with the ropls package using orthoI = NA as the default parameter for datasets with ≥3 biological replicates. Metabolites with VIP > 1 from the OPLS-DA model were pre-selected. Supplementary filtering relied on *p*-value/FDR and fold-change (FC) (valid for ≥2 biological replicates). Features passing both criteria, *p* < 0.05 and VIP > 1.0, were assigned as differential metabolites.

### 2.7. Statistical Analysis

All statistical analysis was conducted with GraphPad Prism Software v.10.1. Data are presented as mean with standard deviation (SD) or median with interquartile range. For comparisons of two or multiple groups, distribution of data was first analyzed by a Shapiro–Wilk test or Kolmogorov–Smirnov test. For normally distributed variables, an unpaired Student’s *t*-test was used for comparisons between 2 groups; one–way ANOVA and Tukey’s post hoc tests were used for comparisons for multiple groups, where the adjusted *p* values were used to determine the significant difference between groups. Pearson correlation analysis was performed to evaluate the cross–chamber molecular correlations between different omics datasets. A *p* value < 0.05 was considered to indicate statistical significance. For multi–omics data, adjusted *p* values < 0.05 were used. For proteomics, differentially expressed proteins (DEPs) were identified with adjusted *p* < 0.05, FC > 1.5 for upregulation and FC < 1.5 for downregulation. For phosphoproteomics, differential phosphosites were defined by adjusted *p* values < 0.05, FC > 1.3 for upregulation and FC < 1.3 for downregulation. For untargeted metabolomics, differential metabolites were screened using adjusted *p* < 0.05, VIP > 1 and |log_2_FC| > 1.

## 3. Results

### 3.1. Cardiac Fibrosis and Tissue Distribution of Dox in Acute and Chronic Rat Models

**Figure 1:**
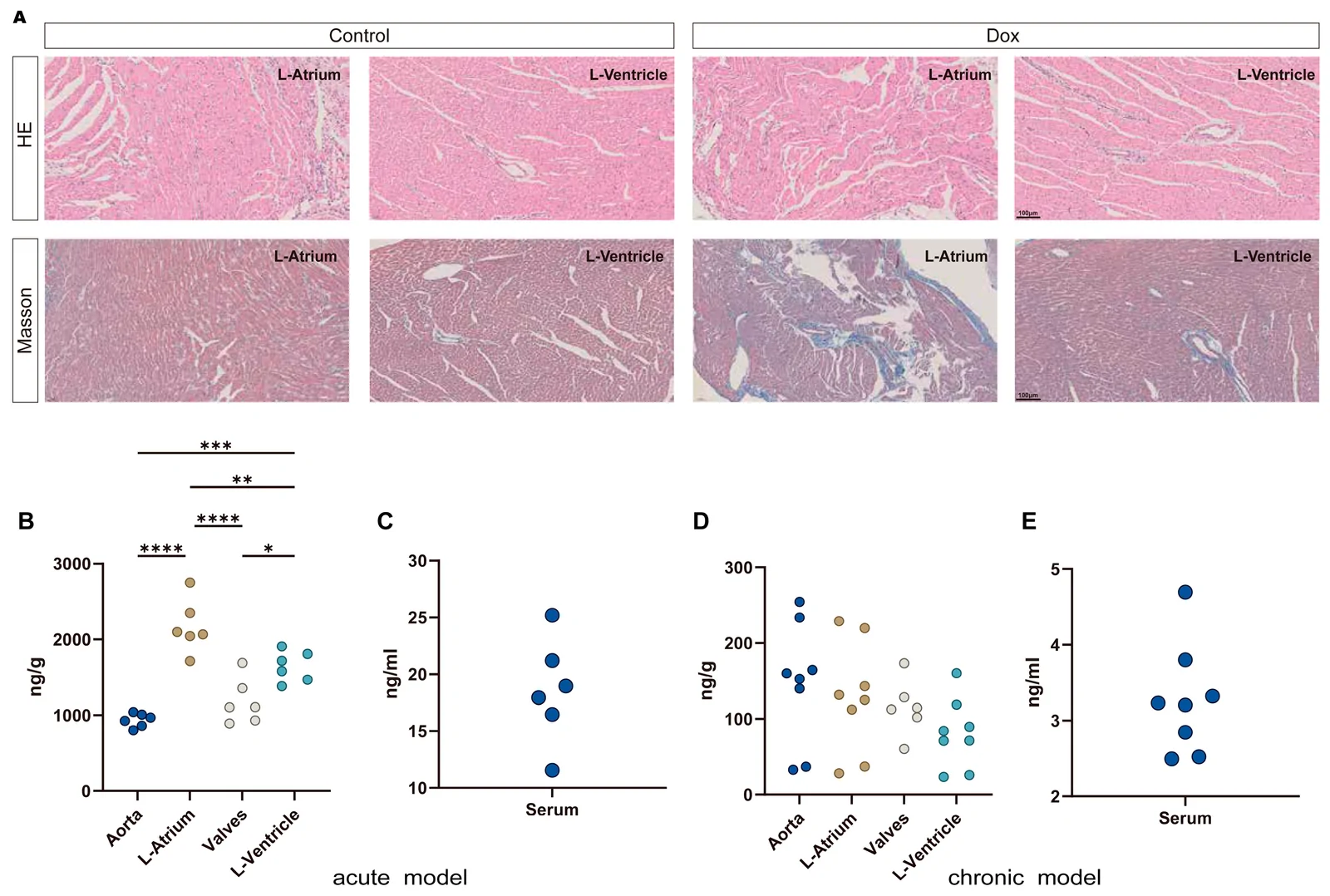
Dox–induced cardiac fibrosis and tissue–specific Dox accumulation in acute and chronic models. (**A**) Representative HE and Masson’s staining of the L–atrium and L–ventricle from the chronic group. For Masson’s staining, dark blue represents fibrotic areas. Scale bar = 100 μm. (**B**) Dox accumulation in the valve, aorta, L–ventricle, and L–atrium from the acute model group; data are presented as median with interquartile range, *n* = 6. (**C**) Serum Dox level in the acute model group; data are presented as mean with SD, *n* = 6. (**D**) Dox accumulation in the valve, aorta, L–ventricle, and L–atrium from the chronic model group; data are presented as median with interquartile range, *n* = 8. (**E**) Serum Dox level in the chronic model group; data are presented as mean with SD, *n* = 8; * *p* < 0.05, ** *p* < 0.01, *** *p* < 0.001, **** *p* < 0.0001.

To validate the cumulative cardiotoxic effects of Dox, we performed HE and Masson’s staining on cardiac tissues from the chronic group rats. In both the L–atrium and L–ventricle, HE staining revealed no marked differences between the control and Dox groups, with no evidence of overt structural disruption or extensive inflammatory cell infiltration. However, Masson’s staining demonstrated pronounced fibrosis in both chambers of the Dox group (Figure 1A). To investigate the accumulation of Dox in different cardiac tissues over time, we measured Dox levels in the valve, aorta, L–ventricle, and L–atrium from both the acute and chronic model groups. The acute model assessed regional drug deposition and early heterogeneous cardiac injury upon short-term Dox exposure, whereas the chronic model examined whether regional drug–accumulation patterns alter with prolonged treatment. Since clinical DIC is primarily a chronic injury, the chronic model was used for subsequent multi–omics profiling. In the acute group, Dox distribution across these regions displayed marked heterogeneity, with the highest accumulation (2171 ± 349 ng/g) in the L–atrium, followed by the L–ventricle, the valve, and finally the aorta (Figure 1B). The mean serum Dox level in the acute group was approximately 18.55 ng/mL (Figure 1C). In contrast, the chronic group exhibited substantially lower Dox accumulation across all regions, with no apparent regional differences (Figure 1D), and the mean serum level declined to 3.266 ng/mL (Figure 1E).

### 3.2. A Chamber-Specific Design Separates Shared Dox Response from Chamber Divergence

**Figure 2:**
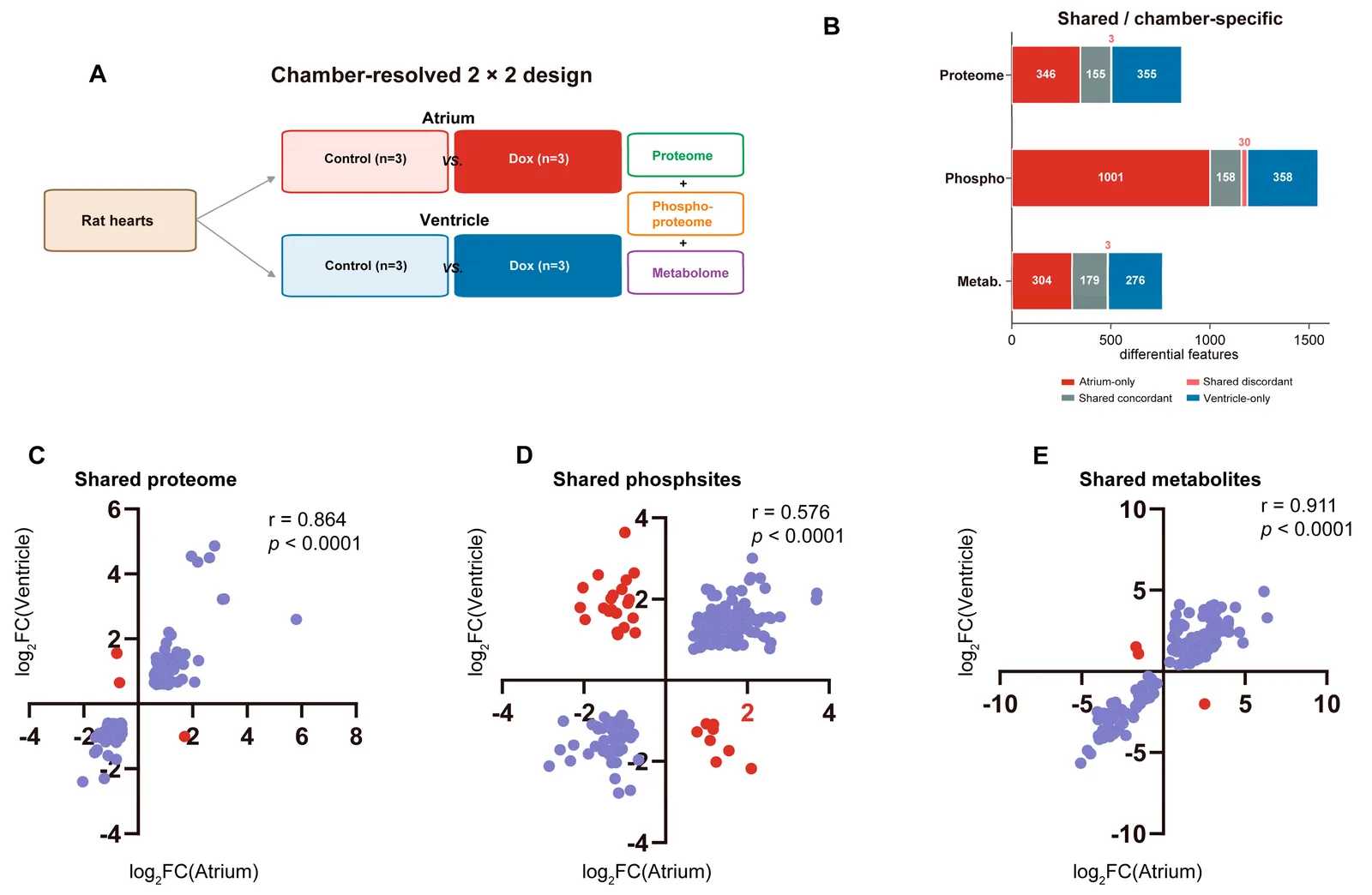
Study design and global response across omics layers. (**A**) Chamber–specific 2 × 2 analytical design. (**B**) Bar chart showing the numbers of differential features in proteome, phosphoproteome and metabolome datasets, grouped by their chamber–dependent alteration patterns. Red: features significantly changed exclusively in the L–atrium; gray–green: common differential features with consistent regulatory directions in both chambers; magenta: common differential features with opposite regulatory directions between L–atrium and L–ventricle; blue: features significantly changed exclusively in the L–ventricle. X–axis: number of differential features; Y–axis: individual omics datasets. (**C**) Scatter plot illustrating cross–chamber log_2_FC correlation of shared DEPs. (**D**) Scatter plot illustrating cross–chamber log_2_FC correlation for shared differential phosphosites. (**E**) Scatter plot illustrating cross–chamber log_2_FC correlation for shared differential metabolites. The X–axis represents log_2_FC values in the L–atrium, and the Y–axis represents log_2_FC values in the L–ventricle. Blue dots denote shared concordant features, which exhibit consistent regulatory directions in both chambers (both upregulated or both downregulated). Red dots denote shared discordant features, showing opposite regulatory directions between the L–atrium and L–ventricle. Pearson correlation coefficient and corresponding *p* value are displayed in the upper-right corner.

The analysis employed a 2 × 2 factorial design, with treatment status and tissue source as the two factors. The four analytical groups were atrium control (control_A), atrium Dox (Dox_A), ventricle control (control_V), and ventricle Dox (Dox_V), each comprising three biological replicates (Figure 2A; Table 1). Differential testing was conducted within each chamber rather than between chambers to ensure that intrinsic baseline differences between chambers were not misinterpreted as treatment effects.

All three molecular layers revealed Dox–related changes. The numbers of differentially abundant proteins and metabolites were comparable between chambers, whereas the phosphoproteomic response was substantially larger in the atrium. Specifically, 1189 differential phosphosites were identified in the atrium, compared with 546 in the ventricle, indicating that site–level regulation represented the most asymmetric layer (Figure 2B; Table 2).

Shared features also exhibited layer–dependent patterns. At the proteome level, 155 of 158 shared proteins changed in the same direction, and the cross–chamber log_2_FC correlation was high (r = 0.864) (Figure 2C). At the metabolome level, 179 of 182 shared metabolic features were concordant (r = 0.911) (Figure 2E). The phosphoproteome displayed the weakest cross–chamber agreement: 30 of 188 shared phosphosites changed in opposite directions, and the correlation was correspondingly lower (r = 0.576) (Figure 2D). Thus, the most pronounced chamber divergence occurred not at the steady–state protein or metabolite level, but at the phosphosite level.

### 3.3. The Proteome Shows a Shared Substrate-Utilization Response with Chamber-Biased Remodeling

**Figure 3:**
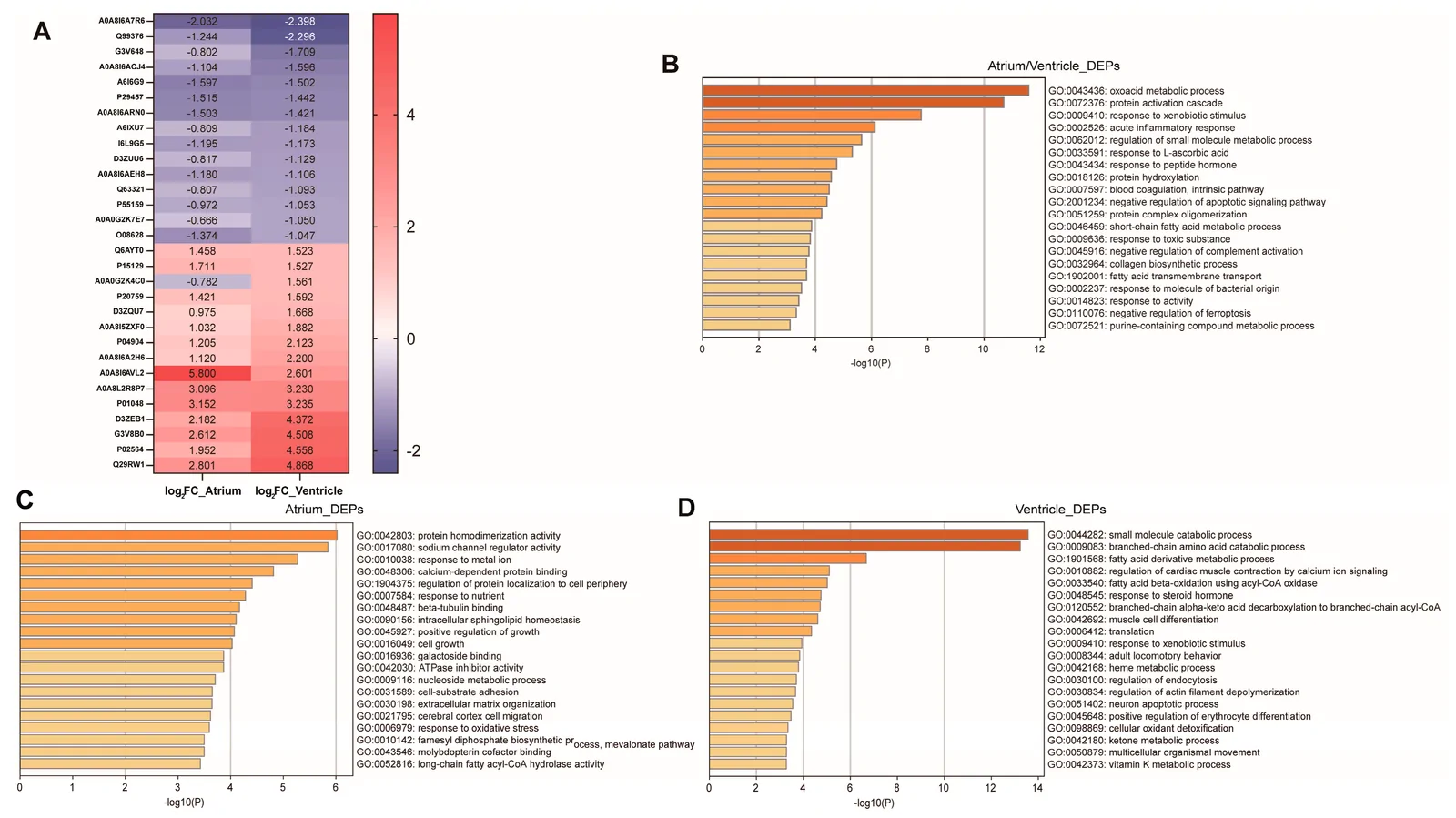
Proteome results. (**A**) The top 30 representative shared differentially expressed proteins (DEPs) between the atrium and ventricle. (**B**) Functional enrichment analysis of shared DEPs. (**C**) Functional enrichment analysis of DEPs exclusively in the atrium. (**D**) Functional enrichment analysis of DEPs exclusively in the ventricle.

At the protein–abundance level, Dox elicited a broad but largely concordant response across chambers. The atrium and ventricle contained similar numbers of differentially abundant proteins (504 and 513, respectively). Among the 158 shared differential proteins, 155 changed in the same direction and only three showed opposite directional changes between chambers. The shared upregulated proteins included CD36, ACOT2, CRAT, HADH, DECR1, ECH1, FMO1, GSTA3 and GCSH. These proteins are linked to fatty acid uptake, acyl–CoA handling, β–oxidation, mitochondrial substrate use and redox adaptation [18,19,20,21]. Shared downregulated proteins included TFRC, AGPAT3, P4HA2 and SERPINH1, indicating that not all components of the common response were increased (Figure 3A).

Functional enrichment analysis revealed that these shared differential proteins were enriched in response to the xenobiotic stimulus, acute inflammatory response, and regulation of small–molecule metabolic process, suggesting a common response to Dox treatment in the atrium and ventricle (Figure 3B). Compared with the shared proteomic response, the chamber–specific differential proteins were more indicative of the heterogeneity between the atrium and ventricle. Functional enrichment analysis of proteins differentially expressed exclusively in the atrium revealed predominant enrichment in sodium channel regulator activity, response to metal ion, and calcium–dependent protein binding, terms largely associated with ion channels and electrical signaling (Figure 3C). In marked contrast, proteins differentially expressed exclusively in the ventricle were predominantly enriched in the branched–chain amino acid catabolic process, fatty acid derivative metabolic process, regulation of cardiac muscle contraction by calcium ion signaling, and fatty acid β–oxidation using acyl–CoA oxidase, terms closely associated with energy metabolism and contractile function (Figure 3D). These results indicate that, at the protein expression level, although the atrium and ventricle exhibit a concordant response to Dox, functionally distinct abnormalities remain clearly distinguishable, likely reflecting their inherent differences in tissue structure and function.

### 3.4. The Phosphoproteome Is the Major Chamber-Divergent Regulatory Layer

**Figure 4:**
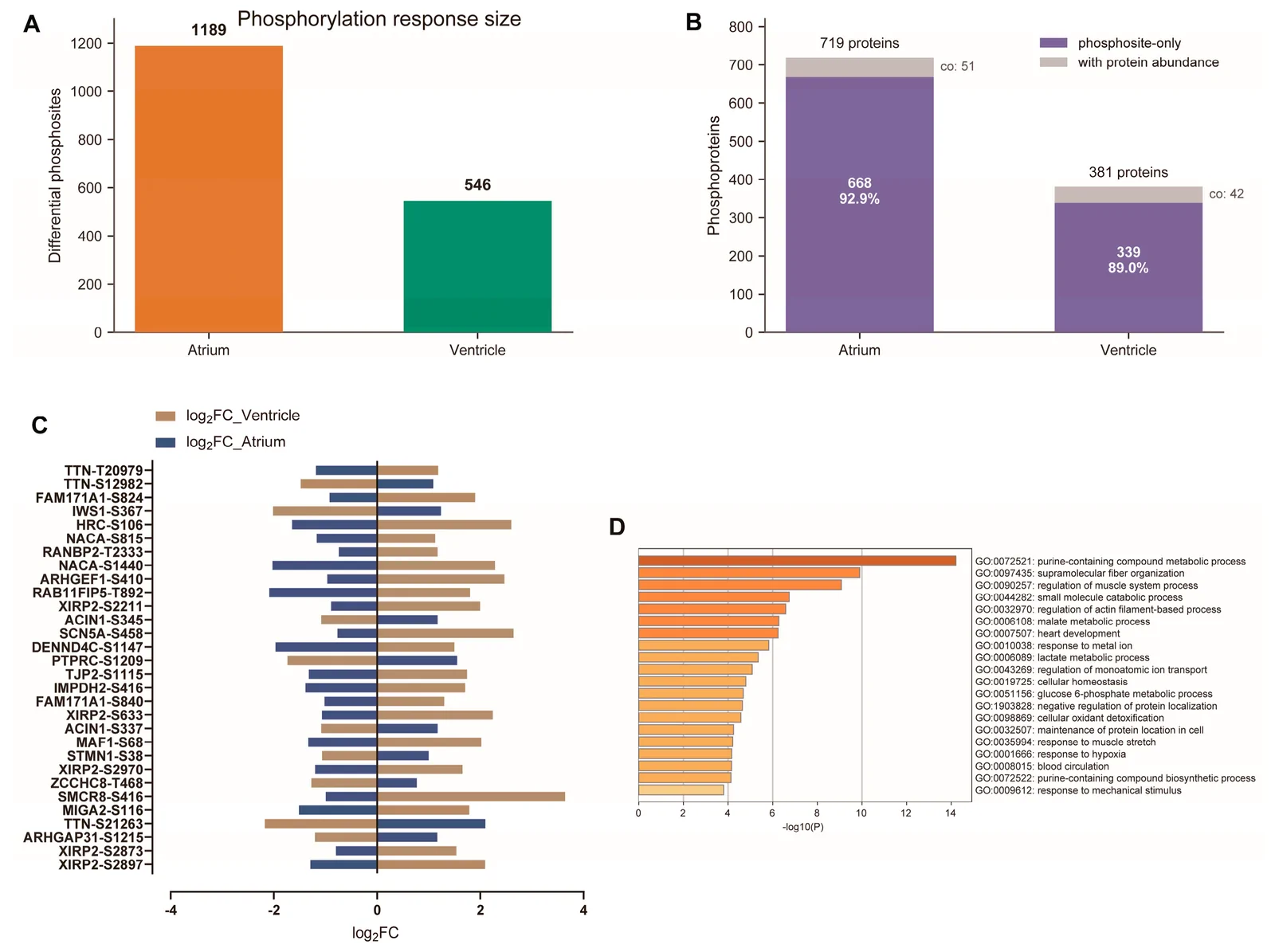
Phosphoproteome results. (**A**) Differential phosphosite counts. (**B**) Shared and chamber–specific phosphosite distribution. (**C**) Representative chamber–biased and opposite–direction phosphosites. (**D**) Functional enrichment analysis of proteins with chamber–biased and opposite-direction phosphosites.

The phosphoproteome differed from the total proteome in both scale and directionality. Differential phosphosites were more numerous in the L–atrium than in the L–ventricle (Figure 4A). This pattern was consistent with the lower cross–chamber correlation observed for phosphosites.

Mapping phosphosites back to their parent proteins revealed that most site–level changes were not accompanied by corresponding changes in total protein abundance. In the atrium, 719 proteins harbored at least one differential phosphosite, yet only 51 of these also showed changes at the protein–abundance level. In the ventricle, the corresponding numbers were 381 and 42. Thus, 92.9% of atrial and 89.0% of ventricular phosphoproteins represented site–only events (Figure 4B; Table 3). These findings indicate that abundance-only profiling would miss the majority of candidate regulatory changes.

Among them, the 30 shared phosphosites with opposite directional changes likely represent key drivers of the divergent responses to Dox between the atrium and ventricle (Figure 4C). Functional enrichment analysis revealed that these sites were predominantly associated with regulation of muscle system process and heart development, which may stem from developmental differences between the two chambers (Figure 4D).

### 3.5. The Metabolome Defines a Shared Lipid and Cofactor Response

**Figure 5:**
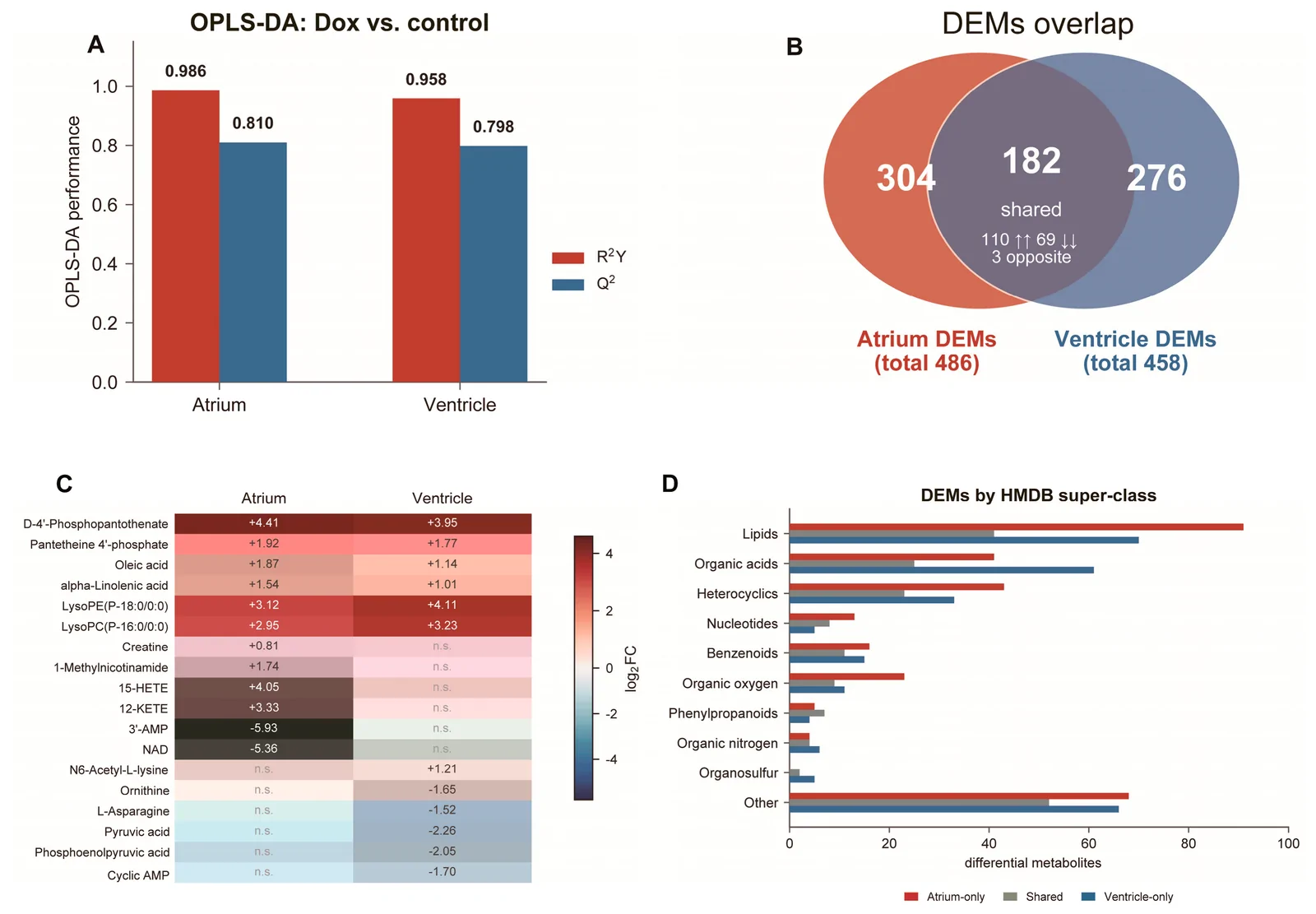
Untargeted–metabolome results. (**A**) OPLS–DA metrics for Dox versus control in each chamber. (**B**) Overlap of differential metabolic features; shared features are separated into 110 increased in both chambers, 69 decreased in both chambers and 3 opposite–direction changes. The upward arrows indicate upregulation, and the downward arrows indicate downregulation. (**C**) Representative metabolites grouped as shared increase, atrial bias and ventricular bias. Non–significant metabolites are shown as n.s. (**D**) HMDB super–class distribution of atrium–only, shared and ventricle-only metabolites.

The metabolome more closely reflected a common Dox response than the phosphoproteome did. The atrium contained 486 differential metabolic features and the ventricle contained 458. OPLS–DA model parameters were high in both chambers (atrium: R^2^Y = 0.986, Q^2^ = 0.810; ventricle: R^2^Y = 0.958, Q^2^ = 0.798), although the small sample size warrants cautious interpretation and targeted validation (Figure 5A).

Among the 182 shared differential metabolites, 179 changed in the same direction. Specifically, 110 features were increased and 69 were decreased in both chambers, with only three features showing opposite directional changes (Figure 5B). Shared increases included D–4′–phosphopantothenate, pantetheine 4′–phosphate, oleic acid, alpha–linolenic acid, LysoPE(P–18:0/0:0), and LysoPC(P–16:0/0:0). This pattern is consistent with alterations in CoA–related metabolism, fatty acid availability, and membrane lipid remodeling [19,22]. Chamber–biased metabolites provided additional discrimination between the two chambers. Atrial–biased changes included increases in creatine, 1–methylnicotinamide, 15–HETE, and 12–KETE, along with decreases in 3′–AMP and NAD. Ventricular-biased changes included an increase in N6–acetyl–L–lysine and decreases in ornithine, L–asparagine, pyruvic acid, phosphoenolpyruvic acid, and cyclic AMP (Figure 5C). HMDB super–class counts showed that lipids and organic acids accounted for a large fraction of chamber–biased metabolic features (Figure 5D).

### 3.6. Multi-Omics Integration Separates Shared and Distinct Metabolic States from Chamber-Specific Regulation

**Figure 6:**
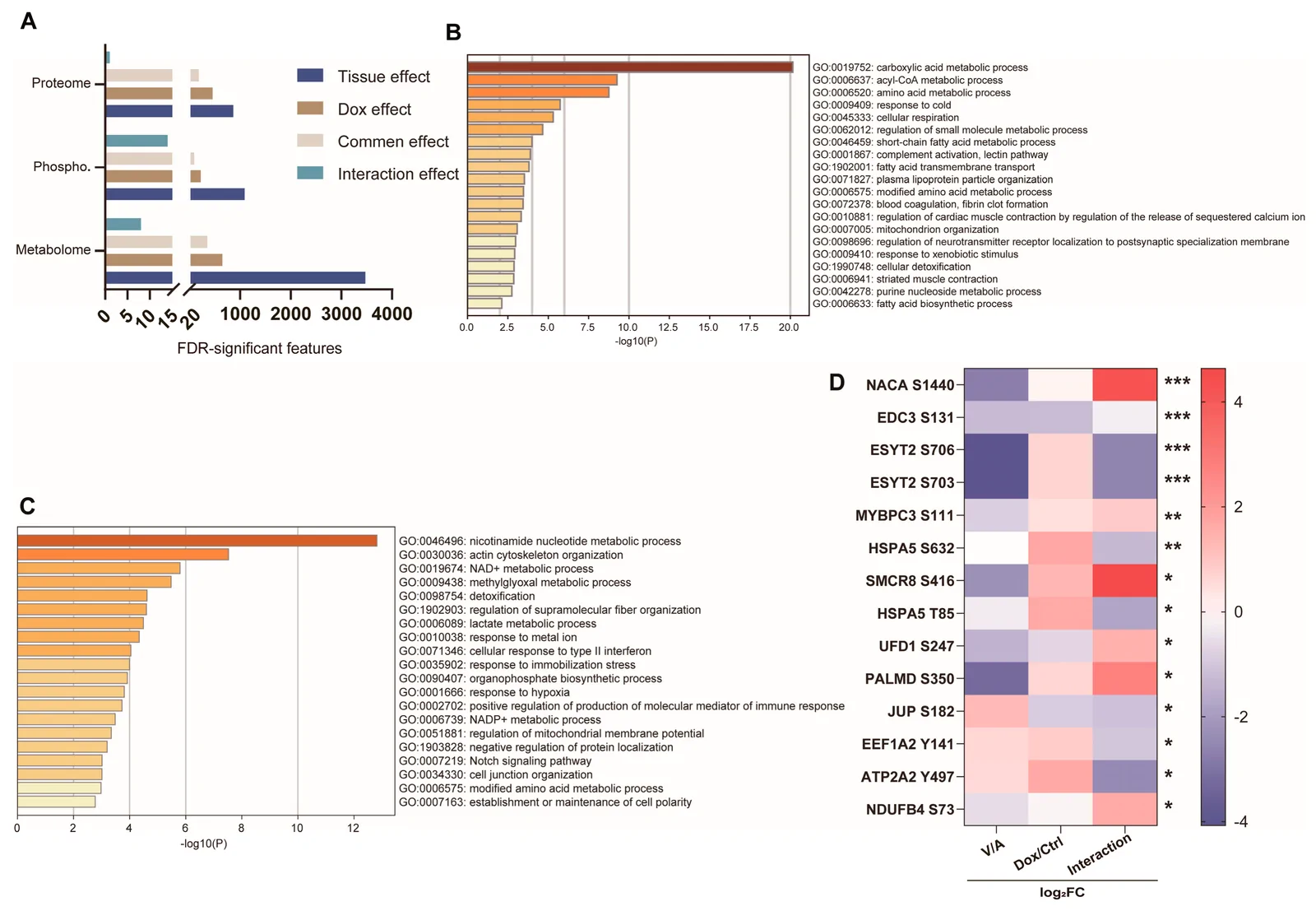
Multi–omics integration. (**A**) Exploratory 2 × 2 decomposition of multi–omics. (**B**) Functional enrichment analysis of DEPs from common effect. (**C**) Functional enrichment analysis of differential expressed phosphoproteins from common effect. (**D**) Heatmap showing candidate phosphosites from interaction effect. All sites exhibited significant interaction effects; * *p* < 0.05, ** *p* < 0.01, *** *p* < 0.001.

A 2 × 2 contingency analysis integrating the multi–omics datasets revealed that tissue–of–origin differences between the atrium and ventricle contributed more pronounced discriminatory features. The common effect of the three omics layers was relatively comparable, reflecting the intrinsic changes induced by Dox. Notably, the phosphoproteome exhibited the most prominent interaction effect, indicating a chamber-differential impact of Dox on the atrium and ventricle (Figure 6A). Functional enrichment analysis of the DEPs from the common effect identified by proteomics revealed associations with the carboxylic acid metabolic process, acyl–CoA metabolic process and amino acid metabolic process, suggesting tissue–concordant effects of Dox that are independent of chamber identity (Figure 6B). Functional enrichment analysis of the differential expressed phosphoproteins from the common effect identified by phosphoproteomics revealed associations with the nicotinamide nucleotide metabolic process, actin cytoskeleton organization and the NAD^+^ metabolic process (Figure 6C). Together with the proteomic findings, these results suggest that Dox induces pan–cardiac metabolic dysregulation. The interaction effects effectively captured the divergent responses of the atrium and ventricle to Dox treatment. Fourteen differential phosphosites identified by interaction effect may mediate the distinct injury responses between the two chambers and thus represent promising candidates for validation (Figure 6D).

## 4. Discussion

In clinical practice, DIC can present with a spectrum of symptoms, including atrial fibrillation [23], bundle branch block [24], and cardiac enlargement progressing to heart failure [25]. These clinical manifestations are likely attributable to both the region–specific accumulation of Dox in the heart and the differential susceptibility of various cardiac structures to the Dox. Our multi–omics dissection of DIC across cardiac chambers reveals three principal findings that extend the current understanding of this complex pathology. First, Dox accumulation is spatially heterogeneous, with the left atrium exhibiting the highest acute drug burden. Second, while the proteomic and metabolomic responses are largely concordant between chambers, the phosphoproteome emerges as the dominant layer of chamber–divergent regulation, with the atrium displaying a more extensive and distinct phosphorylation remodeling. Third, integrative analysis identifies a core set of phosphosites that may serve as molecular switches underlying chamber–specific injury susceptibilities, particularly those linked to metabolic adaptation, ion handling, and cytoskeletal dynamics.

Dox accumulation exhibits regional disparity under acute exposure, with the highest drug levels observed in the left atrium. This phenomenon cannot be fully explained by differences in blood flow or tissue perfusion alone. We hypothesize that such asymmetric drug distribution may result from atrial–specific expression of drug transporters including OATP1A2 and OCT transporters, as well as variations in capillary density and interstitial binding affinity [26]. The marked attenuation of regional differences in the chronic model, accompanied by a sharp decline in serum drug levels, suggests that tissue clearance and redistribution processes gradually homogenize the overall cardiac burden. Nevertheless, the enduring molecular scars, particularly at the phosphosite level, persist and may underpin the progressive nature of DIC even after drug washout.

The phosphoproteome–centric divergence between chambers constitutes the most compelling mechanistic insight from our study. The fact that over 90% of differential phosphosites occurred independently of total protein abundance changes underscores the limitations of relying solely on transcriptomic or proteomic profiling to capture the acute signaling pathway in DIC. The atrium, which harbored more than twice the number of differential phosphosites compared with the ventricle, appears to be a hyper–responsive node in terms of kinase/phosphatase signaling networks. This is particularly intriguing given that the total proteome and metabolome responses were quantitatively similar between the two chambers. We interpret this as evidence that the atrial myocardium, with its distinct embryological origin and thinner wall structure, may rely more heavily on post–translational regulation to cope with oxidative and electrophysiological stresses [27]. The functional enrichment of atrial-biased phosphoproteins in sodium channel regulator activity and calcium–dependent protein binding reinforces this notion, as these processes are directly linked to action potential maintenance and arrhythmogenesis–clinical features that are overrepresented in anthracycline–treated patients [23].

The metabolomic layer, in contrast, provides a unified picture of Dox–induced metabolic disorder. The shared increases in CoA–related intermediates (D–4′–phosphopantothenate, pantetheine 4′–phosphate), free fatty acids (oleic acid, alpha–linolenic acid), and lysophospholipids point to a common disruption in mitochondrial β–oxidation and membrane remodeling [28]. This is consistent with the canonical view of Dox as a mitochondrial poison, but our data demonstrate that this metabolic signature is not chamber–restricted. Instead, it represents a global cardiac stress response that may be triggered by the ubiquitous presence of topoisomerase IIβ in mitochondria across all myocardial cell types [29]. The chamber–biased metabolites, such as atrial–specific increases in creatine and 1–methylnicotinamide, and ventricular–specific decreases in ornithine and pyruvic acid, offer additional discriminatory power and may reflect local differences in energy shuttling, redox balance, and anaplerotic flux. Notably, the decrease in NAD and increase in 15–HETE in the atrium suggest a more pronounced oxidative stress and inflammatory response in this chamber, which could synergize with the phosphoproteomic changes to lower the threshold for atrial arrhythmias [30].

The integrative 2 × 2 decomposition analysis revealed that the common effect of Dox across chambers is predominantly metabolic, whereas the interaction effect is overwhelmingly phosphosite–driven. This hierarchical organization provides a conceptual framework for understanding how a Dox can produce diverse clinical phenotypes. The 14 candidate phosphosites identified from the interaction effect are particularly attractive for future validation, as they represent nodal points where chamber-specific signaling cascades diverge.

Functional mapping of the proteins harboring these 14 phosphosites uncovers a multi–hub regulatory network. ESYT2 (S706 and S703) are involved in membrane contact site formation and lipid metabolism regulation, suggesting that chamber–specific differences may involve phosphorylation–mediated modulation of endoplasmic reticulum–plasma membrane junctions and phospholipid remodeling [31]. MYBPC3 (S111), a critical phosphorylation site on myosin–binding protein C, directly contributes to the maintenance of sarcomeric structure and regulation of contraction and relaxation; its differential phosphorylation between the atrium and ventricle may explain the distinct diastolic functional responses of the two chambers to Dox [32]. ATP2A2 (Y497) may affect sarcoplasmic reticulum calcium reuptake efficiency, and its functional alteration has been demonstrated to be closely associated with the occurrence of atrial fibrillation [33]. Notably, HSPA5 carries two differential phosphosites, S632 and T85, suggesting that the atrium and ventricle differ fundamentally in their activation thresholds or adaptive strategies for the unfolded protein response (UPR) to endoplasmic reticulum stress [34].

This study has several limitations that should be acknowledged. First, each experimental group included only three biological replicates for multi–omics analyses, which may limit statistical power and increase the risk of false–positive or unstable molecular alterations. Future studies with larger sample sizes are needed to validate the observed molecular changes. Second, this study was performed exclusively in male rats. Given known sex–dependent biological responses to doxorubicin–mediated cardiotoxicity in animal models [35], our findings may not be fully generalized to female animals. Third, this study focused exclusively on molecular profiling and did not perform electrophysiological recordings or arrhythmia phenotyping. Accordingly, the associations between atrial molecular remodeling and arrhythmogenic susceptibility remain speculative. The proposed molecular clues for doxorubicin–related atrial dysfunction require further functional and electrophysiological verification to establish direct causal evidence.

## 5. Conclusions

Using chamber–specific bulk multi–omics in rat DIC models, we found that Dox preferentially accumulated in the L–atrium under acute exposure, while regional drug differences vanished after chronic treatment. Proteomic and metabolomic responses were largely consistent between the L–atrium and L–ventricle, whereas the phosphoproteome represented the primary layer driving chamber-divergent injury, with extensive atrial phosphorylation remodeling independent of total protein abundance. Fourteen core phosphosites with significant tissue–drug interaction effects were identified as key candidate mediators.

## Figures and Tables

**Table 1:** Sample design and primary comparisons.

Tissue	Control	Dox-Treated	Primary Comparison
Atrium	*n* = 3	*n* = 3	Dox_A vs. Control_A
Ventricle	*n* = 3	*n* = 3	Dox_V vs. Control_V

**Table 2:** Differential features, shared directions and cross–chamber correlations.

Layer	Atrium	Ventricle	Shared	Concordant	Opposite	r
Proteome	504	513	158	155	3	0.864
Phosphoproteome	1189	546	188	158	30	0.576
Metabolome	486	458	182	179	3	0.911

**Table 3:** Phosphosite-to-protein mapping.

Chamber	Proteins with Differential Sites	Also Differential at Protein Level	Site-Only Proteins	Site-Only Fraction
Atrium	719	51	668	92.9%
Ventricle	381	42	339	89.0%

## Data Availability

The data that support the findings of this study are available from the corresponding authors upon reasonable request.

## Abbreviations

The following abbreviations are used in this manuscript:

Dox	Doxorubicin
DIC	Doxorubicin-induced cardiotoxicity
LA	Left atrium
LV	Left ventricle
LC–MS/MS	Liquid chromatography–tandem mass spectrometry
FC	Fold-change
DEPs	Differentially expressed proteins
DEMs	Differential metabolites
HMDB	Human Metabolome Database
UPR	Unfolded protein response
